# A Ternary Brain-Computer Interface Based on Single-Trial Readiness Potentials of Self-initiated Fine Movements: A Diversified Classification Scheme

**DOI:** 10.3389/fnhum.2017.00254

**Published:** 2017-05-24

**Authors:** Elias Abou Zeid, Alborz Rezazadeh Sereshkeh, Benjamin Schultz, Tom Chau

**Affiliations:** ^1^Bloorview Research Institute, Holland Bloorview Kids Rehabilitation HospitalToronto, ON, Canada; ^2^Institute of Biomaterials and Biomedical Engineering, University of TorontoToronto, ON, Canada

**Keywords:** BCI, EEG, readiness potential, self-initiated fine movement, spatio-temporal filtering, diversified classification scheme

## Abstract

In recent years, the readiness potential (RP), a type of pre-movement neural activity, has been investigated for asynchronous electroencephalogram (EEG)-based brain-computer interfaces (BCIs). Since the RP is attenuated for involuntary movements, a BCI driven by RP alone could facilitate intentional control amid a plethora of unintentional movements. Previous studies have mainly attempted binary single-trial classification of RP. An RP-based BCI with three or more states would expand the options for functional control. Here, we propose a ternary BCI based on single-trial RPs. This BCI classifies amongst an idle state, a left hand and a right hand self-initiated fine movement. A pipeline of spatio-temporal filtering with per participant parameter optimization was used for feature extraction. The ternary classification was decomposed into binary classifications using a decision-directed acyclic graph (DDAG). For each class pair in the DDAG structure, an ordered diversified classifier system (ODCS-DDAG) was used to select the best among various classification algorithms or to combine the results of different classification algorithms. Using EEG data from 14 participants performing self-initiated left or right key presses, punctuated with rest periods, we compared the performance of ODCS-DDAG to a ternary classifier and four popular multiclass decomposition methods using only a single classification algorithm. ODCS-DDAG had the highest performance (0.769 Cohen's Kappa score) and was significantly better than the ternary classifier and two of the four multiclass decomposition methods. Our work supports further study of RP-based BCI for intuitive asynchronous environmental control or augmentative communication.

## Introduction

Brain computer interfaces (BCIs) facilitate direct communication between the brain and external devices, potentially providing people with severe disabilities alternative communication and mobility (Ortiz-Rosario and Adeli, 2013; Burns et al., 2014). Due to its non-invasiveness, high temporal resolution, relative low cost, and convenient operation, electroencephalography (EEG) is the most widely used brain monitoring technique in BCI research. Often, EEG-based BCIs use motor-imagery for target selection such as in wheelchair control (Müller-Putz and Pfurtscheller, 2008; Rodrıguez-Bermudez et al., 2013), and visual (Farwell and Donchin, 1988; Jin et al., 2014) or auditory (Lopez-Gordo et al., 2012; Yin et al., 2016) evoked potentials for communication through on-screen keyboards and spellers. Most of these BCIs require training and are paced by the system rather than the user.

Kornhuber and Deecke (1965) were the first to report the pre-movement neural activity in EEG. This preparatory activity is a slow negative potential that can start as early as 1.5 s before voluntary movement (Kornhuber and Deecke, 1965). This potential is commonly known as the Bereitschaftspotential or readiness potential (RP). The initial segment of the RP (known as the “early RP”) is slow-evolving and symmetric over the central-medial cortex (Shibasaki and Hallett, 2006). The potential becomes lateralized (known as the “late RP”) ~500 ms prior to movement onset, with a steeper negative slope and larger amplitude over the contralateral primary motor cortex (Shibasaki and Hallett, 2006). An RP-based BCI would offer asynchronous control allowing users to execute actions at will rather than according to system-paced cues. However, being a slow non-oscillatory cortical potential and occurring concurrently with task-unrelated brain activity, the RP is typically not visible in single trials and is, therefore challenging to detect. Nonetheless, two recent studies of single-trial RP signals demonstrated the detection of self-paced fine movement (Abou Zeid and Chau, 2015) and arm reaching movement (Lew et al., 2012) intention from rest state. The lateralized RP has also been exploited in combination with imagined movement rhythms to improve the speed and accuracy of BCIs (Blankertz et al., 2003, 2006). Other studies have used the RP to distinguish the laterality (i.e., left or right hand) of an upcoming movement (Blankertz et al., 2001, 2011; Wang et al., 2004; Liao et al., 2007; Tomioka and Müller, 2010; Lu et al., 2014; Abou Zeid et al., 2016). RP-based BCIs have been also investigated in patients with amyotrophic lateral sclerosis (Kübler and Birbaumer, 2008) and stroke patients (Jankelowitz and Colebatch, 2005; Muralidharan et al., 2011).

The majority of RP-based BCI studies have investigated a binary classification problem such as detecting movement intention from a rest state (Lew et al., 2012; Abou Zeid and Chau, 2015) or laterality prediction of movement (Blankertz et al., 2001, 2011; Wang et al., 2004; Liao et al., 2007; Tomioka and Müller, 2010; Lu et al., 2014; Abou Zeid et al., 2016). Limited work has considered the differentiation of more than two states. In Jochumsen et al. (2013), authors achieved average classification accuracy of 59.25% between four different levels and speeds of intended right ankle movements. Another study (Jochumsen et al., 2015) showed the possibility of classifying between three different grasp tasks with an accuracy of 63%. A most recent study (Hassan et al., 2015) showed that, following detection of movement from rest, an average classification accuracy of 73% can be achieved between two different right ankle movements. However, none of these studies considered fine movements.

In the literature, a classification challenge that involves more than two classes is labeled as a multiclass problem. Because of their complex decision boundaries, multiclass classifiers are usually more difficult to build than binary classifiers (Galar et al., 2011). This is why decomposition techniques are widely used to divide the original multiclass problem into easier to solve binary classifications. Two common decomposition techniques are the one-against-one (OAO) and one-against-all (OAA) (Lorena et al., 2008; Rokach, 2010). The OAO builds a binary classifier for each possible class pair, and the outputs of these binary classifiers are combined to predict the output class. A disadvantage of OAO is that each classifier is exclusively trained on data from two classes; during testing, when the classifier is exposed to instances from a previously unseen class the output would be non-competent and could negatively affect the final classification results (Furnkranz et al., 2009). The OAA builds a binary classifier for each class, separating it from the all other classes. A disadvantage of OAA is that imbalanced training datasets may be produced when instances from a single class are compared to all other instances in the dataset, causing undesirable effects in the derived classifiers (Sun et al., 2009). Both OAO and OAA require methods that combine the outputs of the binary classifiers to produce the final result. The most common methods for combining outputs in OAO are voting (Friedman, 1996), weighted voting (Hüllermeier and Vanderlooy, 2010), and probability estimates (Wu et al., 2004). Maximum confidence and dynamic ordering (Hong et al., 2008) combination methods are used for OAA. Alternatives to OAO and OAA are the hierarchical strategies (Lorena et al., 2008), which, in a tree structure format, perform successively more refined discrimination until the final class is obtained. Two well-known hierarchical strategies are the directed binary tree (DBT) (Schwenker and Palm, 2001) and the decision directed acyclic graph (DDAG) (Platt et al., 2000). Each node in the DBT corresponds to a binary classifier that distinguishes between two sets of classes. With the DDAG, however, each node corresponds to a binary classifier for one pair of classes. Initially all classes are candidates, and according to the root classifier, one of the classes is eliminated and a new node is defined. This process is iterated until a leaf node is reached corresponding to the output class. One disadvantage of the DDAG is that the results may depend on the sequence of the binary classifiers in the nodes of the graph (Kijsirikul and Ussivakul, 2002). Hierarchical strategies do not require a separate method of combining classifier decisions. Therefore, they can naturally deal with test cases in the unclassifiable region of OAO, where ties in voting occur.

Although other motor related EEG signals, notably the event-related desynchronization (ERD) (Liao et al., 2007), have been studied in BCI, RP remains unique: RP and ERD reflect different neurophysiological phenomena of the sensorimotor cortex (Pfurtscheller and Da Silva, 1999). RP is specific to voluntary movement (Obeso et al., 1981; Baker et al., 2012), whereas ERD occurs with voluntary, passive and imagined movements (Formaggio et al., 2013). The RP may thus serve as an identifying cue for voluntary control in the presence of excessive unintentional movements, such as those characteristic of athetoid cerebral palsy (Hou et al., 2006). In addition, the RP associated with extant fine movements may be particularly informative for those with severe disabilities, due to, for example, cervical spinal cord injury (Blain et al., 2010) or degenerative neuromuscular conditions (Power and Chau, 2013), where only residual motor activity is retained. Moreover, a RP-based BCI with three or more states would expand the options for functional control beyond that of a binary system. In this paper, we propose, single-trial classification amongst three states: a clear idle (rest) state, and RP signals preceding a self-initiated left or right fine motor movement. We evaluate our method on a multi-subject dataset.

## Methods

### Experimental setup

**Figure 1:**
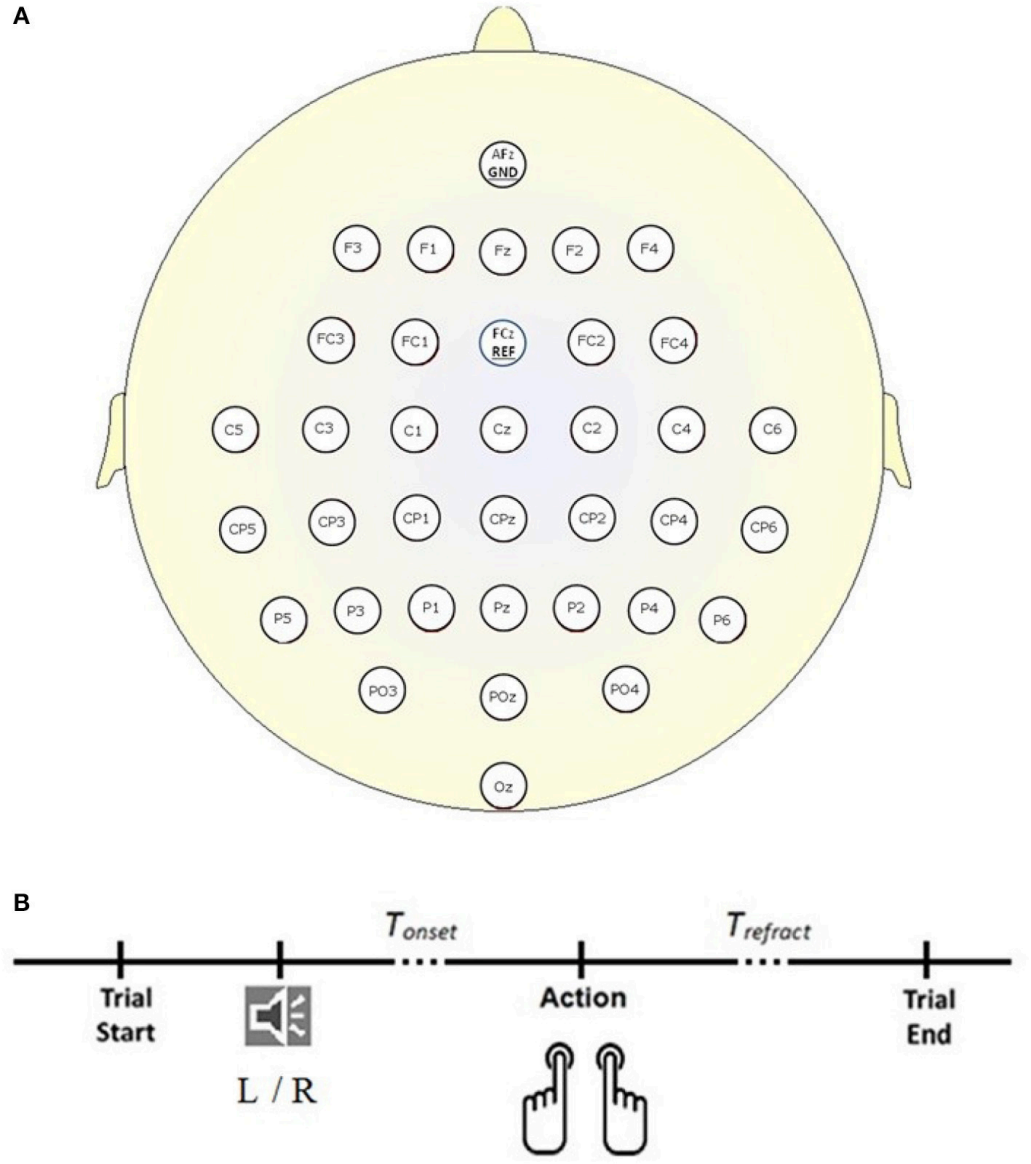
**Experimental setup. (A)** Locations of the 34 EEG electrodes. **(B)** Timeline of a trial. Each trial began in the idle state where the participant rested his or her forearms, and elbows on the table, with the index fingers of the left and right hand on the key “D” and “L” respectively. A “left” (L) or “right” (R) auditory cue informed the participant that a self-initiated keystroke could be made. *T*_*refract*_ is a refractory period of 4 s following the keystroke and before the start of the next trial.

The dataset (Abou Zeid et al., 2016), was collected according to the protocol shown in Figure 1B. Briefly, 34 channels of EEG signals were recorded at 1 kHz from 14 able-bodied participants (P1–P14; 25.57 ± 5.03 years; three male; two left-handed), at the scalp locations depicted in Figure 1A, using a BrainAmp actiCap (Brain Products) during self-initiated keyboard presses (letters “D” and “L”) with their left and right index fingers. In each trial, participants heard the word “left” or “right” in a female voice. Instead of reacting to the cue, participants were asked to press the key with the corresponding finger on their own time. For each participant, 400 trials (200 left and 200 right) were recorded. Trials where the keystroke occurred within 2 s of the auditory cue (i.e., reactive and not a self-initiated response) were discarded. Trials where multiple key presses occurred were also discarded. On average, 390 trials were retained and post-auditory cue of 4.76 ± 1.98 s elapsed before participants pressed the key (*T*_*onset*_ in Figure 1B). Four channels of electrooculography (EOG) signals were also collected at 1 kHz to facilitate the suppression of eye movement artifacts in the EEG. For each trial, the 500 ms interval preceding the auditory cue was taken as the rest signal, while the 500 ms interval ending 130 ms before the key press was extracted as the RP signal. For further information and justification of the experimental setup and dataset, please refer to Abou Zeid et al. (2016).

**Figure 2:**
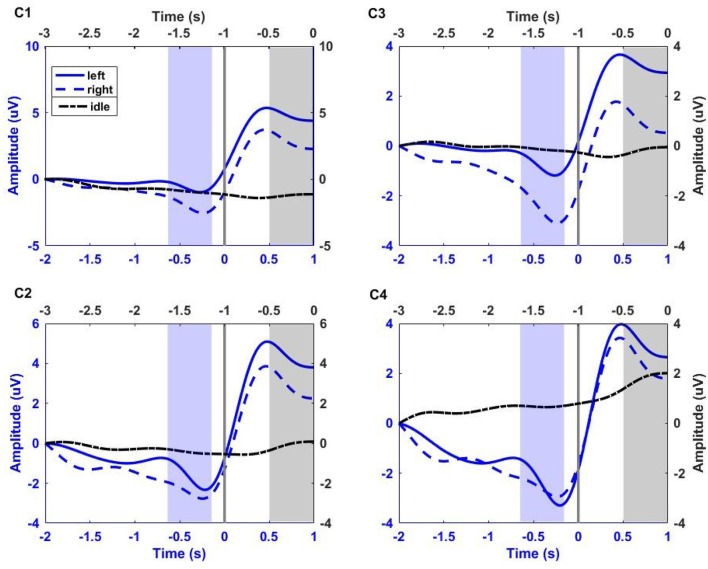
**Group trial average of left and right RP period (corresponding to [−2 1] s with respect to key press, blue x-axis), and idle period (corresponding to [−3 0] s before the auditory cue, black x-axis) for all participants at electrodes C1, C2, C3, and C4**. The gray vertical line at 0 s, on blue x-axis, corresponds to the key press. EEG signals were filtered between 0 and 1 Hz, and baseline corrected. Solid blue line, dashed blue line, and dash-dotted black line correspond to left and right hand fine movement, and idle period respectively. The shaded blue region (ending 130 ms before key press) and shaded black region (ending just before auditory cue) correspond to the 500 ms interval of analysis for movement and idle states respectively.

For illustration, Figure 2 shows the low-pass filtered (0–1 Hz) EEG signals from channels C1, C3, C2, and C4 averaged over all trials and for all participants. The idle (dash-dotted black line) interval corresponds to [−3 0] s before the auditory cue. The left (solid blue line) and right (dashed red line) RP intervals correspond to [−2 1] s with respect to the time of key press (Time = 0 s). The shaded black region corresponds to the 500 ms segment of idle state analysis. The shaded violet region corresponds to the 500 ms segment of RP analysis, ending 130 ms before key press. This interval of analysis includes the RP peak negativity and a clear laterality of the RP (i.e., more pronounced negativity on the side contralateral to movement), especially for channel C1 and C3.

### Feature extraction

**Figure 3:**
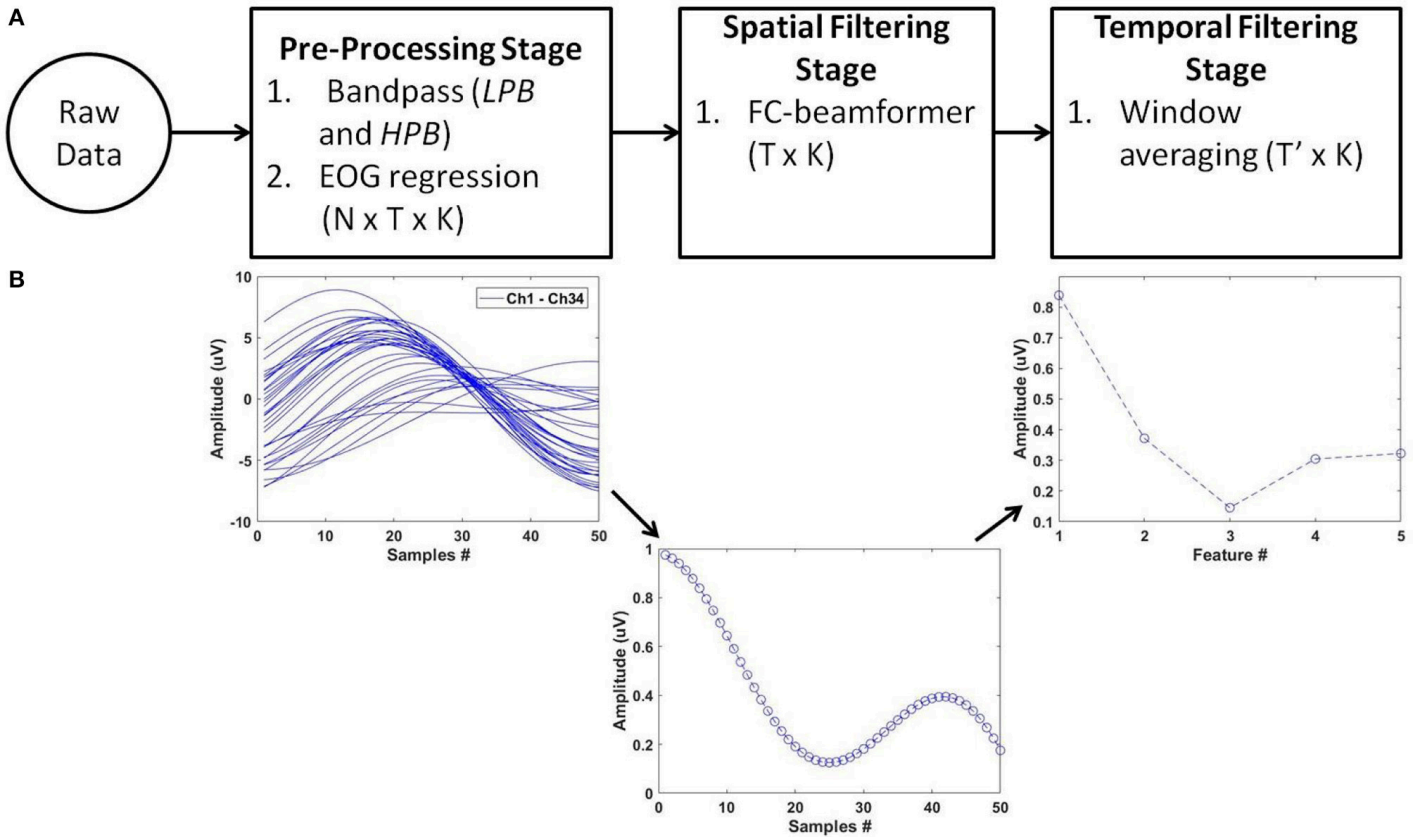
**The PSTF feature extraction algorithm. (A)** Flowchart: *N* is the number of channels, *T* is the number of data samples and *K* the total number of trials. Raw data of dimensions *N* × *T* × *K* are pre-processed by a low-pass (LPB as cut-off) and a high-pass (HPB as cut-off) filters. EOG regression is applied for eye artifact removal. The spatial filtering stage applies a Fisher criterion (FC) beamformer to reduce the feature dimensions to *T* × *K*. The temporal filtering stage applies non-overlapping window averaging to reduce feature dimensionality to *T*′ × *K*, with (*T* ′≪ *T*). LPB, HPB, and the window averaging size are determined by optimization on training data on a per participant basis. **(B)** Processing of a single trial (*N* = 34; *T* = 50) along the PSTF stages.

For feature extraction, we deployed the pipeline of spatio-temporal filtering (PSTF) algorithm (Abou Zeid et al., 2016), which was previously introduced for binary classification. The PSTF derives discriminatory features based on participant-specific optimization of temporal and spatial filtering of signals from all available EEG electrodes. Figure 3 summarizes the stages of the algorithm in a flowchart and shows an example of the signal pattern produced after each stage. In this paper, we briefly describe the mathematical formulation of the PSTF and its extension to multi-category classification.

The pre-processing stage consisted of participant-specific low- and high-pass filters that maximized participant-specific classification accuracy on training data. Eye movement artifacts were suppressed via EOG regression (Schlögl et al., 2007). Next, the pre-processed signals were then filtered using a Fisher criterion (Bishop, 2006a) beamformer, determined with regularization. Such spatial filtering increased the separation between classes while minimizing the variance within a class. Thus, the objective was to maximize the Fisher criterion quotient in Equation (1):

(1) $$J\left(W\right)=\frac{W^{\prime }S_{b}W}{W^{\prime }S_{w}W}$$

where *S*_*b*_ and *S*_*w*_ are the spatial between-class and within-class covariance matrices, respectively. Hence, the optimal spatial filter *W* is found by solving the eigenvalue problem in Equation (2) (provided *S*_*W*_ is non-singular):

(2) $$(S_{w}{\;}^{-1}S_{b})W=W\Lambda$$

where Λ is a matrix of eigenvalues. Taking the spatio-temporal matrix *X*_*i, k*_ (dimension *N* × *T*, where *N* is the number of channels and *T* the number of data samples) from each trial *k* of class *C*_*i*_, the matrices *S*_*b*_ and *S*_*w*_ are computed by Equations (3) and (4):

(3) $$S_{b}=\underset{i=1}{\sum^{N_{c}}}p_{i}\left(M_{i}-M\right){\left(M_{i}-M\right)}^{\prime }$$

(4) $$S_{w}=\underset{i=1}{\sum^{N_{c}}}\underset{k=1}{\sum^{K_{i}}}(X_{i,k}-M_{i})(X_{i,k}-M_{i}{)}^{\prime }$$

Where *K*_*i*_ is the number of trials belonging to class *C*_*i*_. *N*_*c*_ is the number of classes. In case of ternary classification, these are *C*_1_, *C*_2_, and *C*_3_ representing, respectively, the left movement, right movement, and idle classes. The symbol *p*_*i*_ is the class probability. The average of the trials in class *C*_*i*_ and of all trials are denoted by *M*_*i*_ and *M* respectively, and computed by Equations (5) and (6):

(5) $$M_{i}=\frac{1}{K_{i}}\underset{k=1}{\sum^{K_{i}}}X_{i,k}$$

(6) $$M=\frac{1}{K}\underset{k=1}{\sum^{K}}X_{k}$$

where *K* is the total number of trials, and *X*_*k*_ is the spatio-temporal data matrix.

Finally, the selected filter *W*^(1)^ (*N* × 1) is the eigenvector associated with the largest eigenvalue of $\left({S_{W}}^{-1}S_{b}\right)$. For a given matrix *X*_*k*_ (dimension *N* × *T*), the spatial filter output *y*_*k*_ (1 × *T*) is given by Equation (7):

(7) $$y_{k}=\;W^{{\left(1\right)}^{\prime }}X_{k}$$

Since *W* is estimated from noisy EEG signals, to improve generalization (i.e., reduce noise overfitting, and enforce non-singularity), regularization may be used. This is achieved by replacing *S*_*w*_ by [*S*_*w*_ + γ*I*] in Equation (4), where γ > 0 and *I* is a correspondingly sized identity matrix. The regularized parameter γ was determined by applying cross-validation on training data.

Lastly, the temporal filtering stage consisted of signal averaging over consecutive non-overlapping windows. This filtering captures the temporal evolution of the RP while reducing the feature dimensionality. Such reduction in dimension is preferred for classification given the limited size of our dataset.

### Multiclass classification

We attempted to solve the ternary classification problem (left movement vs. right movement vs. idle state) directly through a multiclass classifier (MCS), and, via the following binary decomposition techniques: OAO, OAA, DBT, and DDAG.

#### MCS

The PSTF was used in its ternary form to produce training features for the three classes. These features were fed to a classifier to estimate the boundaries that best separate the three classes. The estimated PSTF and classifier parameters were applied on the test cases to predict their labels.

#### OAO

**Figure 4:**
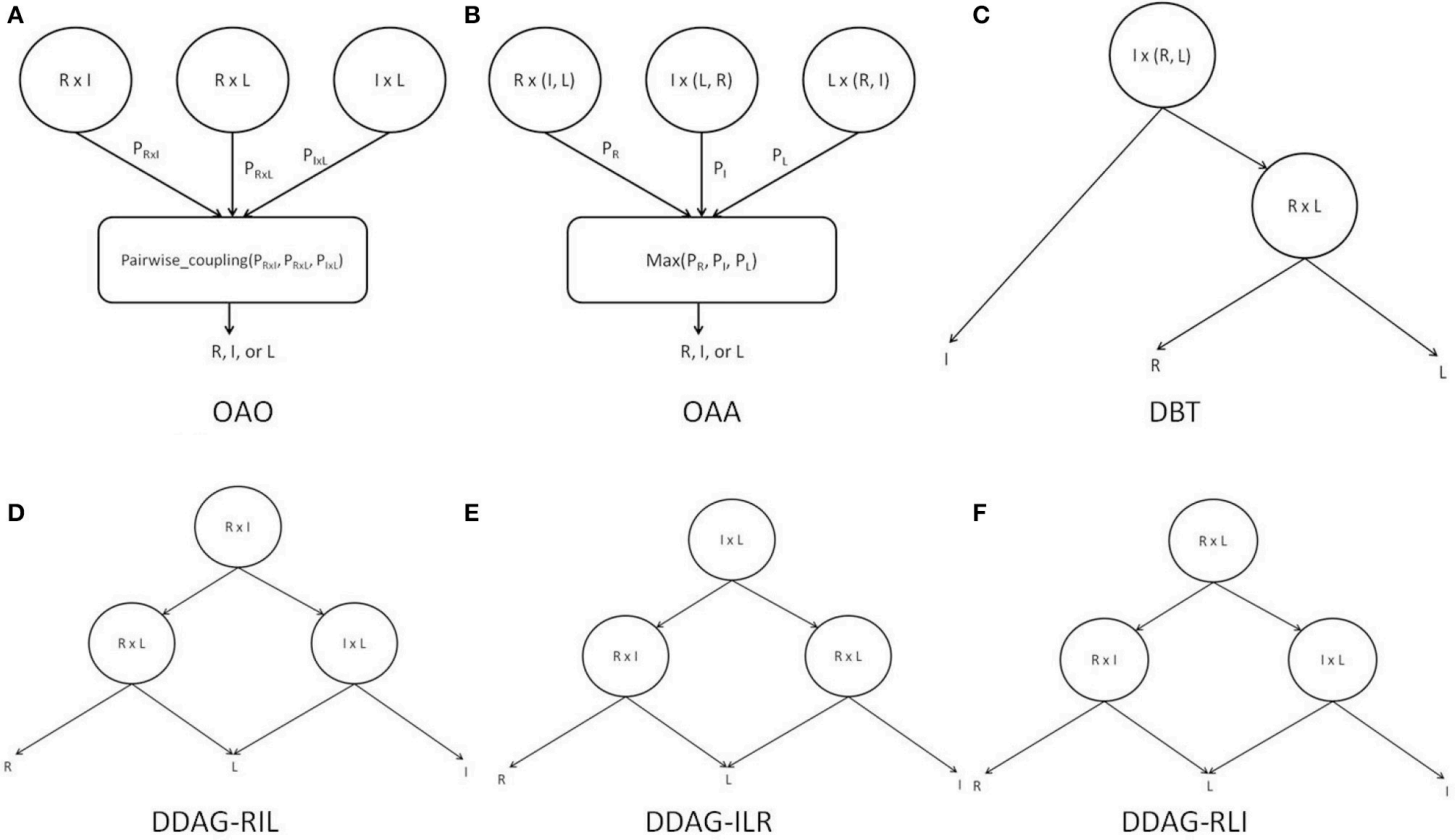
**Binary decomposition techniques: (A)** OAO with pairwise-coupling combination; **(B)** OAA with maximum probability combination; **(C)** DBT structure; **(D)** DDAG RIL structure; **(E)** DDAG ILR structure; **(F)** DDAG RLI structure. “I” refers to idle class, “L” refers to left movement class, and “R” refers to right movement class.

The PSTF was used in its binary form to provide training features for three different binary classifiers: R-I (right vs. idle), L-I (left movement vs. idle), and R-L (right movement vs. left movement). Each of the classifiers was trained independently and its boundaries and parameters estimated. Each test case was presented to each classifier and all class probabilities were retained. Individual classifier probabilities were combined by the pairwise coupling algorithm (Wu et al., 2004) to infer the label for the test case (Figure 4A).

#### OAA

The PSTF was used in its binary form to provide training features for three different binary classifiers: R-A (right movement vs. All {idle, left movement}), I-A (idle vs. All {left movement, right movement}), and L-A (left movement vs. All {right movement, idle}). Each of the classifiers was trained independently and each had its boundaries and parameters estimated. Each test case was presented to every classifier and all class probabilities were retained. The predicted label was the class with the highest probability across classifiers (Figure 4B).

#### DBT

The PSTF was used in its binary form to provide training features for two different classifiers: I-(L, R) (idle vs. left/right movement), L-R (left vs. right movement). Each classifier was trained independently and its boundaries and parameters estimated. A test case was passed through the DBT structure in Figure 4C, representing an intuitive approach for solving the multiclass problem. The root node performed a general discrimination between the idle or movement (i.e., left/right movement) classes. If a test case was classified as movement, a subsequent node refined the decision as being either a left or right movement.

#### DDAG

The PSTF was used in its binary form to provide training features for three different binary classifiers. Each classifier was trained independently and its boundaries and parameters estimated. We distinguished among three different DDAG structures as shown in Figures 4D–F. A test case was passed through a DDAG starting from the root node. According to the result of the root node classifier, one of the classes was eliminated and a second node distinguished between the remaining two classes. Due to different permutations of nodes in the graph, the three distinct DDAGs may yield different results, as highlighted in Kijsirikul and Ussivakul (2002).

### Ordered diversified classifier system

A multiple classifier system is an ensemble of competent and diversely trained classifiers (Lysiak et al., 2014) with the aim of improving classification accuracy (Xu et al., 1992; Ho et al., 1994; Kittler et al., 1998). While classifier diversity can be achieved through a variety of strategies (Kang et al., 2015), the most successful employs different classification algorithms. Indeed, the best classification algorithm may vary within a dataset, from participant to participant and even from data sample to data sample (Woods et al., 1997; Cavalin et al., 2013).

We propose an ordered diversified classifier system (ODCS) for the aforementioned multiclass problem. The principle behind the ODCS is to determine the best combination of classification algorithms for every sub-problem derived from binary decomposition of the original multiclass problem. A similar approach has been presented in Kang et al. (2015) but is specific to OAO and is limited to identifying the best single classification algorithm (rather than classifier combination). In contrast, ODCS can be used with any binary decomposition technique and admits the best combination of any number of classifiers according to a competence-ordered list of the candidate classification algorithms. The overall ODCS procedure as described in Algorithm 1 can be summarized as follows. Consider a training dataset of *N* data samples, *D* = {*X*_*i*_, *y*_*i*_|*i* = 1, …, *N*}, where *X*_*i*_ is a feature vector and *y*_*i*_ ∈ {*C*_1_, …, *C*_*c*_} denotes its class label. For a class pair (*j, k*), we form the subset *D*_*jk*_ of *D* that consists of the data samples belonging to the jth or kth classes. Next, we build the candidate classifiers Γ_*A*_1_, *D*_*jk*__, …, Γ_*A*_*M*_, *D*_*jk*__ with the subset *D*_*jk*_ using a pre-defined set of classification algorithms {*A*_1_, …, *A*_*M*_}. For each candidate classifier, validation accuracy is computed on a subset of training data and the candidate classifiers are ordered by decreasing performance (i.e., validation accuracy). This classifier ordering is performed on every class pair in the binary decomposition algorithm (i.e., DDAG, OAO, etc.). The classification result from the ordered classifiers can then be used in a combination strategy to produce the final classification result.

Two general types of classifier combination strategies can be used, yielding different multiple classifier systems: classifier selection and classifier fusion (Kuncheva, 2002b). Classifier selection uses the best classification algorithm, whereas classifier fusion combines the outcome of multiple classification algorithms through weighting. Classifier selection performs well in cases where a single algorithm is superior to all others. In contrast, classifier fusion is preferred when the classification algorithms perform comparably, and the group consensus may improve the overall accuracy.

Among the early methods of classifier fusion (Kittler et al., 1998; Kuncheva, 2002a), none considered classifier reliability. One of the first to do so, Ahangi et al. (2013) proposed a weighted majority voting, in which the vote of each classifier was weighted by a measure of its performance. Nikjoo et al. (2011) suggested a reputation-based classification in which the decision of each classifier was weighed on the basis of its past performance. In this work, we use the reliability weighted average (RWA) technique (Abou Zeid and Chau, 2015). It weights the classification algorithms by their validation accuracies. For a data pattern *Z*, the fused posterior probability for a class *C*_*j*_ given *M* classifiers as in (8):

(8) $$P\left(C_{j}|Z\right)=\underset{i=1}{\sum^{M}}\omega_{i}p(C_{j}|X_{i})$$

where *X*_*i*_ is the feature vector representation of pattern *Z* for the i^th^ classifier, ω_*i*_ is the weight for classifier *i*: $\omega_{i}=\frac{e^{VA_{i}}}{\sum_{j=1}^{M}e^{VA_{j}}}$, with *VA*_*i*_ being the validation accuracy of classifier *i*. Finally, the data pattern *Z* is assigned to the class with highest posterior probability.

**Algorithm 1:** Ordered diversified classifier system (ODCS).

**Input**: training dataset *D* = {*X*_*i*_, *y*_*i*_|*i* = 1, …, *N*},
*y*_*i*_ ∈ {*C*_1_, …, *C*_*c*_}, candidate classifier algorithms *A*_1_, …, *A*_*M*_
**Output**: ordered classifier set Γ_*jk*_ for every class pair (*j, k*)
** procedure ODCS**
** for** each class pair (*j, k*) do
- *D_jk_* ← set of data samples whose class labels are *j* or *k*
- train the different candidate classifiers
Γ_*A*_1_,*D*_*jk*__, …, Γ_*A*_*M*_,*D*_*jk*__
- obtain validation accuracy for each candidate classifier
- Γ*_jk_* ← Γ*_A_*1,*_Djk_*, …, Γ*_AM_*,*_Djk_* ordered by descending
validation accuracy
** end for**
**end procedure**

### Implementation

For PSTF implementation, the data were filtered bi-directionally (zero-phase shift) by IIR Butterworth low- and high-pass filters. The candidate frequency cut-offs were {1, 2, 3, 4, 5, 6, 7 Hz} for the low-pass filter, and {none, 0.1, 0.3, 0.5} for the high-pass filter. For the FC beamformer, the candidate regularization coefficients were {0, 1, 10, 10^3^, 10^4^, 10^5^, 10^6^}. In the temporal filtering stage, the candidate window sizes were {5, 10, 25}. The optimal combination of frequency cut-off, regularization coefficient, and time window size were determined by five-fold cross validation on training data for each participant.

Each trial from the experimental protocol, presented in Experimental setup above, was composed of an idle period and either a left or a right movement period. This protocol produced more samples of the idle class than left or right movement classes. Therefore, for classifier training, where applicable, the idle class was subsampled to create a balanced training set. Also, due to limited number of available trials (average of 195 left or right movement valid trials per participant), the dataset was split into 10 equal and distinct chunks, 9 used for training and 1 for testing. Testing was repeated 10 fold, each time using a distinct chunk of data.

As candidate classification algorithms for the ODCS, five well-known classification algorithms were selected (Bishop, 2006a,b): linear probabilistic Gaussian model (LPGM), support vector machines (SVM), Fisher linear discriminant analysis (FLDA), logistic regression (LR), and regularized least square (RLS). The following is a brief description of these algorithms. In this paper, the ODCS was applied with each class pair in the DDAG structure for either the selection of the best classification algorithm (ODCS1-DDAG) or fusion of the results of the best two (ODCS2-DDAG), three (ODCS3-DDAG), four (ODCS4-DDAG), and five (ODCS5-DDAG) classification algorithms.

LPGM models the training features by unimodal Gaussian distributions, and assumes that classes share a common covariance matrix, leading to linear decision boundaries. This assumption limits model complexity and improves generalization by reducing the risk of noise over-fitting. Following the estimation of the class-conditional Gaussian distributions from training data, Bayes' theorem is used to infer the posterior probability of a test case belonging to a certain class. We presented a complete mathematical formulation of LPGM in Abou Zeid and Chau (2015).

SVM is a linear classifier that seeks to find the maximum margin hyperplane that separates one class from another (Bishop, 2006b). It can deal with non-linear classification problems by mapping the input space through kernel functions to a higher dimensional space. SVM formulates a convex optimization problem that can be solved through sequential minimal optimization. The radial basis function (RBF) kernel was used.

FLDA projects the input features onto a one-dimensional space that maximizes class separation. The projection vector is computed following the Fisher's criterion (Bishop, 2006a) by maximizing the between-class variance while minimizing the within-class variance. A discriminant is computed on the projected data by using LPGM.

LR forms a logistic function of a linear combination of the input variables whose output is in the range of [0, 1]. The best weight of the combination can be estimated by maximizing the likelihood function on the training data and using gradient decent methods (Bishop, 2006a).

RLS estimates the linear model (i.e., weights) associated with each of the classes by minimizing the sum-of-squared error function on training data (Bishop, 2006a). A regularizer λ was used to limit the growth of the weights, which facilitated model training with a modestly sized data set while mitigating the risk of severely over-fitting the model to noise. For λ, the candidate values were {0, 0.5, 1, 10, 10^2^, 10^3^, 10^4^, 10^5^ }. The optimal λ was determined by 5 fold cross-validation on training data for each individual participant.

### Performance evaluation

As described in Section Implementation above, the dataset was unbalanced (i.e., unequal number of samples per class due to the nature of the experiment). To measure the agreement between the predicted and desired selections in the presence of unbalanced data, Cohen's kappa (*k*) (Cohen, 1960; Thomas et al., 2013) coefficient was used instead of accuracy rate. *k* was computed from the confusion matrix as in (9):

(9) $$k=\frac{N\sum_{i=1}^{m}h_{ii}-\sum_{i=1}^{m}T_{ri}T_{ci}}{N^{2}-\;\sum_{i=1}^{m}T_{ri}T_{ci}}$$

where *h*_*ii*_ are the main diagonal elements of the confusion matrix (i.e., the number of true positives for each class), (Tex translation failed) is the number of examples, *m* is the number of class labels, and *T*_*ri*_ and *T*_*ci*_ are the marginal row and column counts, respectively. *k* ranges from −1 (total disagreement) through 0 (chance-level classification) to 1 (perfect agreement).

The main difference between the accuracy rate and *k* is the scoring of correct classifications. The accuracy rate scores all the successes over all classes, whereas *k* scores the successes independently for each class and aggregates them. The *k* measure is less sensitive to randomness and bias caused by unequal numbers of examples in each class, and therefore is the preferred method of evaluation with unbalanced classes (Danker-Hopfe et al, 2004; Galar et al., 2011).

## Results

Table 1 lists the mean of the 10 fold test *k* score over all participants, for various binary decomposition methods (OAA, OAA, DBT, DDAG) and classification algorithms (LPGM, SVM, FLDA, LR, RLS). Among the different classification algorithms, FLDA resulted in the highest *k* score for all binary decomposition methods (OAA-FLDA: 0.735; OAA-FLDA: 0.733; DBT-FLDA: 0.701; DDAG-FLDA: 0.725). The DDAG results shown are from the RIL structure (Figure 4D). There was no significant difference in *k* score between the various DDAG structures (Figures 4D–F).

**Table 1:** **Mean and standard deviation of the 10 fold test Kappa (κ) score across all participants, for the different binary decomposition methods and different classification algorithms**.

**Binary decomposition method**	**LPGM**	**SVM**	**FLDA**	**LR**	**RLS**
**OAO**	0.676 ± 0.116	0.647 ± 0.096	0.735 ± 0.111	0.716 ± 0.14	0.659 ± 0.144
**OAA**	0.672 ± 0.128	0.659 ± 0.098	0.733 ± 0.114	0.708 ± 0.127	0.665 ± 0.13
**DBT**	0.637 ± 0.116	0.606 ± 0.091	0.701 ± 0.105	0.689 ± 0.119	0.639 ± 0.117
**DDAG**	0.672 ± 0.116	0.631 ± 0.099	0.725 ± 0.114	0.708 ± 0.138	0.665 ± 0.126

*OAO, one-against-one; OAA, one-against-all; DBT, directed binary tree; DDAG, decision directed acyclic graph. LPGM, linear probabilistic Gaussian model; SVM, support vector machine; FLDA, Fisher linear discriminant analysis; LR, logistic regression; RLS, regularized least square*.

**Figure 5:**
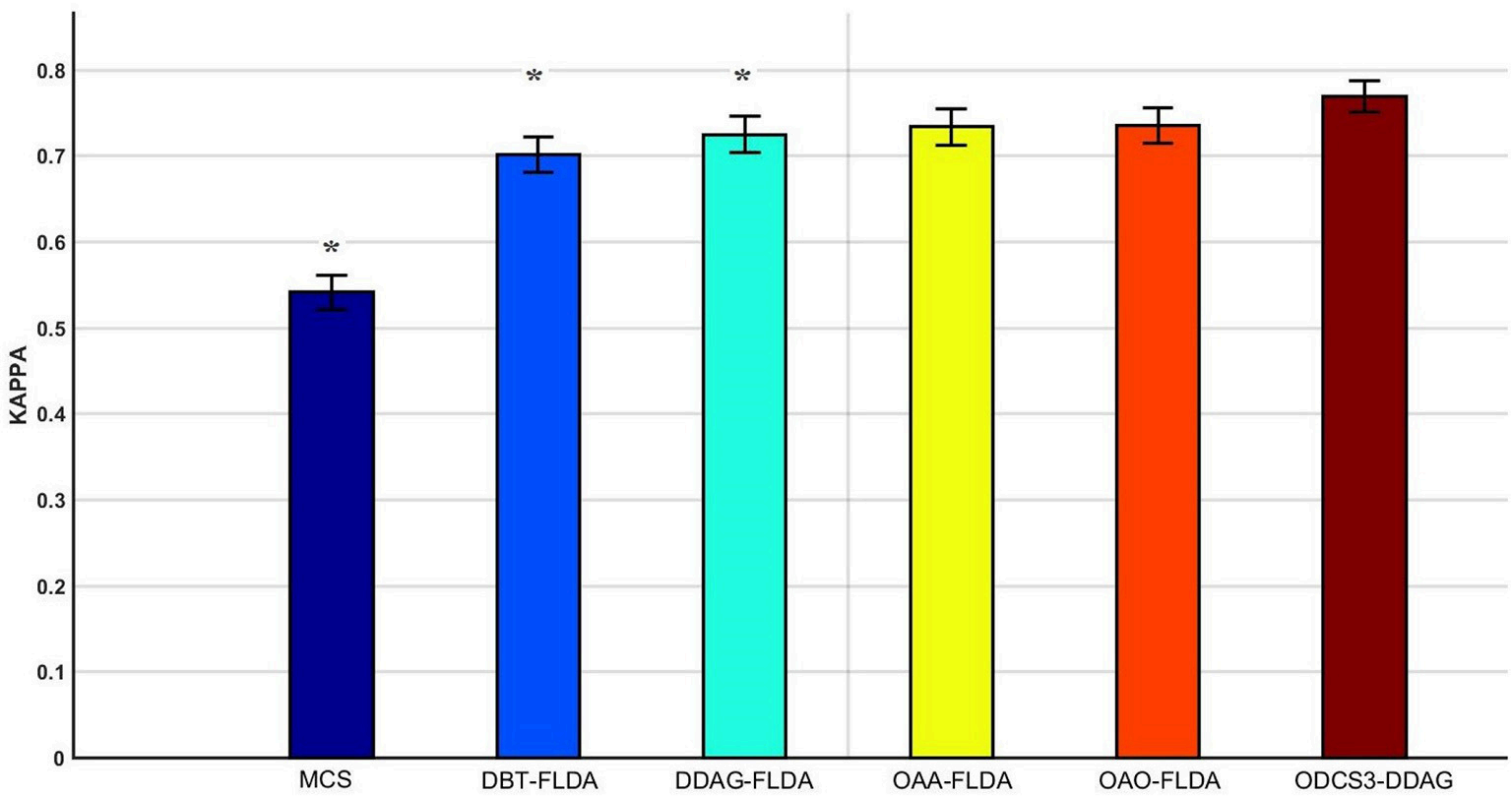
**Mean of the 10 fold test Kappa (κ) score comparisons over all participants and for multiple classifications methods (MCS, multiclass classifier using LPGM classification algorithm; DBT-FLDA, directed binary tree using FLDA classification algorithm; DDAG-FLDA, decision directed acyclic graph using FLDA classification algorithm; OAA-FLDA, one-against-all using FLDA classification algorithm; OAO-FLDA, one-against-one using FLDA classification algorithm; ODCS3-DDAG, fusion of best 3 classification algorithms using ODCS {ordered diversified classifier system} applied with DDAG)**. Five classification algorithms (LPGM, SVM, FLDA, LR, and RLS) have been used in ODCS3-DDAG. Error bars represent the 95% confidence interval computed over all participants. Methods with significant pairwise difference to ODCS3-DDAG are denoted by an asterisk (^*^).

Figure 5 shows significant differences (ANOVA, *p* < 0.05) in the mean 10 fold test *k* scores among the various methods (MCS: 0.541 ± 0.01; DBT-FLDA: 0.701 ± 0.01; DDAG-FLDA: 0.725 ± 0.011; OAA-FLDA: 0.733 ± 0.01; OAO-FLDA: 0.735 ± 0.01; ODCS3-DDAG: 0.769 ± 0.009) when considering all participants. For ODCS3-DDAG, the ODCS method was applied for classifier fusion (i.e., fusion of the results of the best three classification algorithms among LPGM, SVM, FLDA, LR, and RLS) with each class pair in the DDAG structure. *Post-hoc* pair-wise comparison (Table 2), with Bonferroni correction, reveals that ODCS3-DDAG achieves a significantly higher *k* score than MCS (*p* = 2.088 × 10^−46^), DBT-FLDA (*p* = 7.83 × 10^−5^), and DDAG-FLDA (*p* = 0.039). No significant difference in *k* score is found when comparing ODCS3-DDAG to OAA-FLDA (*p* = 0.239) or OAO-FLDA (*p* = 0.329).

**Table 2:** **Multiple comparisons between the various classification methods using the Bonferroni correction**.

**Method1 vs. Method2**	**ODCS3-DDAG**	**OAO-FLDA**	**OAA-FLDA**	**DDAG-FLDA**	**DBT-FLDA**	**MCS**	
ODCS3-DDAG	0	0.329	0.239	0.039	7.84 × 10^−5^	2.088 × 10^−46^	
OAO-FLDA	0.329	0	1.000	1.000	0.333	6539 × 10^−35^	
OAA-FLDA	0.239	1.000	0	1.000	0.454	2.437 × 10^−34^	
DDAG-FLDA	0.039	1.000	1.000	0	1.000	1.489 × 10^−31^	
DBT-FLDA	7.84 × 10^−5^	0.333	0.454	1.000	0	1.160 × 10^−24^	
MCS	2.088 × 10^−46^	6.539 × 10^−35^	2.437 × 10^−34^	1.489 × 10^−31^	1.160 × 10^−24^	0	

*Two methods are significantly different if their comparison p-value is < 0.05*.

The mean of the tenfold test *k* score, across all participants, for classification algorithm selection (ODCS1-DDAG) was 0.735 ± 0.01. The same score was 0.760 ± 0.009, 0.769 ± 0.009, 0.766 ± 0.009, and 0.751 ± 0.010 for the fusion of two, three, four, and five best classification algorithms, respectively. A non-parametric ANOVA test indicated equivalence (*p* = 0.073) between selection and fusion methods.

Table 3 shows the total confusion matrix for ODCS3-DDAG computed from 10 test folds, for all participants.

**Table 3:** **ODCS3-DDAG total confusion matrix across all 10 fold (test sets) and for all participants**.

**Correct class**	**Predicted class**		
	**Right**	**Idle**	**Left**
Right	**1,896 (72.37%)**	116 (4.43%)	608 (23.20%)
Idle	51 (0.97%)	**5,043 (96.24%)**	146 (2.79%)
Left	454 (17.33%)	128 (4.88%)	**2,038 (77.79%)**

*The values in bold correspond to number and percentage of correctly classified instances*.

Table 4 lists the selection percentage (%) for each classification algorithm. The table entries indicate how often ODCS selected each candidate algorithm (columns) as the best for a given class pairing (rows).

**Table 4:** **Percent of time each classification algorithm was selected as the best by ODCS, for each class pairing on training data**.

**Pairs**	**LPGM**	**SVM**	**FLDA**	**LR**	**RLS**
Right vs. Idle	0.0	34.0	28.0	38.0	0.0
Left vs. Idle	0.0	44.0	30.0	26.0	0.0
Right vs. Left	0.0	0.0	4.0	19.0	77.0

## Discussion

Our findings demonstrate the successful differentiation between idle state and an upcoming left or right fine movement from single trial analysis of the RP in a self-initiated key press protocol with able-bodied individuals.

As shown in Table 1, among the employed classification algorithms (LPGM, SVM, FLDA, LR, RLS), FLDA resulted in the highest mean *k* score for all of the binary decomposition methods (OAA, OAA, DBT, and DDAG) with a single classification algorithm. Such result is mainly due to the fact that FLDA projects the input features onto a one-dimensional space that maximizes class separation (Bishop, 2006a).

Overall, the proposed decomposition method (ODCS3-DDAG) yielded significantly higher (*p* < 0.05, Table 2) mean *k* score (0.769 ± 0.009; Figure 5), than the multiclass classifier (MCS: 0.542 ± 0.010) and some of the binary decomposition methods with a single best classification algorithm (DBT-FLDA: 0.701 ± 0.01; DDAG-FLDA: 0.725 ± 0.011). ODCS3-DDAG performance was not significantly better than OAA-FLDA (0.733 ± 0.01; *p* = 0.239) or OAO-FLDA (0.735 ± 0.01; *p* = 0.329). However, ODCS3-DDAG had a higher mean *k* score and a tighter 95% confidence interval that slightly overlapped (Figure 5) with that of OAA-FLDA and OAO-FLDA. Furthermore, for 10 out of 14 participants, ODCS3-DDAG achieved the highest 10 fold average test *k* score. These results corroborate previous reports that group consensus among different classification algorithms tends to outperform an individual classifier (Stashuk and Paoli, 1998; Kamel and Wanas, 2003). Important to mention that ODCS3-DDAG avoids the complexity of the combination methods required by OAO-FLDA (Wu et al., 2004) and OAA-FLDA (Hong et al., 2008), as shown in Figure 4.

### Comparing multiclass and decomposition classification

Figure 5 and Table 2 show that each of the binary decomposition methods (OAO-FLDA: 0.735 ± 0.01; OAA-FLDA: 0.733 ± 0.01; DBT-FLDA: 0.701 ± 0.01; DDAG-FLDA: 0.725 ± 0.011) outperformed the multiclass classifier (MCS: 0.541 ± 0.01) when using as a single classification algorithm (i.e., when ODCS is not applied). This finding is not surprising, as generally, for any classification algorithm, the decision boundaries of a multiclass problem tend to be more difficult to compute than that of a binary classification problem (Galar et al., 2011).

Amongst the binary decomposition methods, when using a single classification algorithm, no significant difference in performance (Figure 5 and Table 2) was observed (OAO-FLDA: 0.733 ± 0.01; OAA-FLDA: 0.733 ± 0.01; DBT-FLDA: 0.701 ± 0.01; DDAG-FLDA: 0.725 ± 0.011). Nonetheless, DBT and DDAG avoid the complexity of the combination methods needed with, namely, OAO (Wu et al., 2004) and OAA (Hong et al., 2008), as shown in Figure 4.

### Comparing classification algorithm selection and fusion

With ODCS, classification algorithm selection (i.e., selection of the best classification algorithm) or fusion (i.e., combination of the results of the best two or more classification algorithms) can be used to produce the final results. Although there was an increase in *k* score for ODCS2-DDAG, ODCS3-DDAG, ODCS4-DDAG, and ODCS5-DDAG compared to classification algorithm selection (ODCS1-DDAG), these differences were not significant. One way to interpret this finding is that the consensus among all classification algorithms may not improve upon the decisions of the best classification algorithm when those decisions are already highly accurate. Indeed, the performance of classifier fusion is never worse than the average of the individual classifiers, but not necessarily better than the best classifier (Stashuk and Paoli, 1998; Kamel and Wanas, 2003).

### Analysis of the confusion matrix

The total confusion matrix in Table 3 shows that ODCS3-DDAG is highly sensitive and specific to the idle class with classification accuracy of 96.24%. The results are less accurate, but well-above chance level, for the right (72.37%) and left class (77.79%), with a slightly better performance for the latter. Most of the right class misses (23.20%) are predicted as left class and vice versa for the left class (17.33%). It is known that a left or right hand movement would activate similar areas in the motor cortex, although with stronger activation in the hemisphere contralateral to the movement (Shibasaki and Hallett, 2006).

### Selection of classification algorithms

As shown in Table 4, the selection of the best classification algorithm varied by class pair. For the right vs. idle class pair, SVM (34%), FLDA (28%), and LR (38%) were the only selected. In the case of left vs. idle, the same three classification algorithms were selected (SVM 44%, FLDA 30%, and LR 26%), with a greater preference for SVM. It is known that the RPs preceding left and right hand movements both arise from the motor cortex, albeit with stronger activation in the contralateral hemisphere (Shibasaki and Hallett, 2006). Therefore, it is conceivable that similarities existed between the classification problems for these two class pairs (right vs. idle and left vs. idle), leading to the selection of the same classification algorithms. For the right vs. left class pair, RLS was predominantly selected (77% of the time), followed by LR (19% of the time), and FLDA (only 4% of the time). This indicates that among the five classification algorithms, RLS is best suited to fine movement laterality prediction. Interestingly, LPGM was never selected, indicating that Gaussian modeling of the feature distributions was not appropriate.

To the best of our knowledge, this paper is the first work on ternary classification of fine movements from RP features. Herein, the achieved results (average *k* score of 0.769% and associated average classification accuracy of 82.13%) are comparable to published results on RP-based binary BCIs: 76% average sensitivity in predicting gross movement from idle state (Lew et al., 2012); 82.21% average classification accuracy in predicting fine movements from idle state (Abou Zeid and Chau, 2015); 76.27% (Lu et al., 2014) and 74.99% (Abou Zeid et al., 2016) average classification accuracies in predicting the laterality of fine movements. Furthermore, the results in this paper are superior to reported performance on multiclass classification of movement related potentials: 59.25% average classification accuracy amongst four different levels and speeds of intended right ankle movements (Jochumsen et al., 2013); 63% average classification accuracy amongst three different grasp tasks (Jochumsen et al., 2015); 73% average classification accuracy amongst two different right angle movements, following detection of movement from rest (Hassan et al., 2015).

Future efforts should focus on enhancing the classification between left and right movements. One approach might be to consider more candidate classification algorithms, especially ones that can model non-linearity (among the algorithms described in this paper, only SVM can deal with non-linearity), such as neural networks (given enough data are available). Ultimately, we would like to evaluate the proposed ternary RP-based BCI with individuals possessing only residual muscle movement, in an online, asynchronous rather than system-paced paradigm. The algorithms described herein are easily trained and calibrated offline. Once calibrated, these algorithms can be implemented by simple operations making it suitable for real-time systems. Since the RP is a pre-motor potential preceding voluntary movement (Shibasaki and Hallett, 2006), eliciting it requires no cue, and it can be detected in a continuous flow of neuronal signal. Indeed, using RP signals, we have shown successful prediction of fine movement from idle state in a simulated online paradigm (Abou Zeid and Chau, 2015). Likewise, the ternary BCI described here should be validated online. Functional contexts for such an online system might include real-time driving of a powered wheelchair or operation of an augmentative and alternative communication aid.

## Conclusions

In this paper, we proposed the ODCS as a classifier selection-fusion algorithm for use with multiclass decomposition methods. We applied the ODCS with a decision directed acyclic graph (ODCS-DDAG). We compared the ODCS-DDAG to a multiclass classifier and other multiclass decomposition methods (OAO, OAA, DBT, DDAG) for ternary classification amongst an idle state, a left movement and a right movement on the basis of readiness potential signals. On a dataset of 14 able-bodied participants, ODCS-DDAG achieved significantly better classification in terms of average Cohen's Kappa score, when compared to ternary classifier, DBT, and DDAG. Average Cohen's Kappa scores did not differ significantly between classification algorithm selection and classification algorithms fusion.

## Ethics statement

This study was carried out in accordance with the recommendations of “Research Ethics Board of Holland Bloorview Kids Rehabilitation Hospital” with written informed consent from all subjects. All subjects gave written informed consent in accordance with the Declaration of Helsinki. The protocol was approved by the “Research Ethics Board of Holland Bloorview Kids Rehabilitation Hospital.”

## Author contributions

EAZ designed the study protocol, participated in data collection, performed the literature review, data analysis, and algorithm implementation, and wrote the manuscript. ARS led the recruitment of participants and the collection of data. BS contributed to the recruitment of participants, collection of data, and performed data validation. TC is the principal investigator. He supervised the work, provided general guidance, reviewed and edited the manuscript.

## Funding

The first author was supported by the following grant: NSERC Discovery Grant RGPIN-2014-06077.

### Conflict of interest statement

The authors declare that the research was conducted in the absence of any commercial or financial relationships that could be construed as a potential conflict of interest.
